# Public health round-up

**DOI:** 10.2471/BLT.18.010218

**Published:** 2018-02-01

**Diphtheria vaccination in Bangladesh**

School children show their vaccination cards after a diphtheria–tetanus immunization campaign in Cox’s Bazar in Bangladesh. Since October 2017, at least 28 deaths and 3014 suspected cases of diphtheria have been reported in Cox’s Bazar, where more than 600 000 Rohingyas have arrived from Myanmar since August. bit.ly/2D3bMFs

**Figure:**
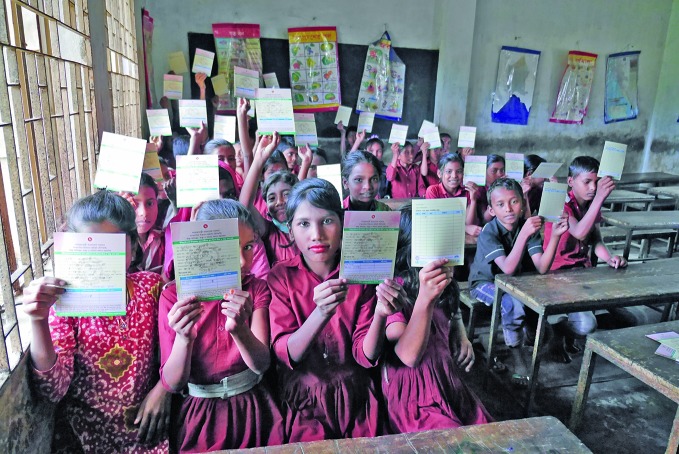
WHO/Dalia Lourenço

## Typhoid vaccine prequalified

WHO has prequalified the first conjugate vaccine to prevent typhoid fever. The vaccine is Typbar-TCV® developed by Indian pharmaceutical company Bharat Biotech.

The vaccine confers long-lasting immunity, requires only one dose and can be given to children as young as six months through routine childhood immunization programmes. 

Other typhoid vaccines are recommended for children over 2 years of age.

Prequalification by WHO means that the vaccine meets standards of quality, safety and efficacy, thus making it eligible for procurement by the United Nations Children’s Fund and other United Nations agencies.

A conjugate vaccine is one that is composed of a polysaccharide antigen that is fused to a carrier molecule.

In October 2017, the Strategic Advisory Group of Experts (SAGE) on immunization, which advises WHO, recommended typhoid conjugate vaccine for routine use in children over six months of age in typhoid endemic countries.

SAGE also called for the introduction of typhoid conjugate vaccine to be prioritized for countries with the highest burden of typhoid disease or of antibiotic resistance to *Salmonella *typhi, the bacterium that causes the disease.

Use of the vaccine should also help to curb the frequent use of antibiotics for treatment of presumed typhoid fever, and thus help slow the increase in antibiotic resistance in *Salmonella* typhi. 

Shortly after SAGE’s recommendation, the board of Gavi, the Vaccine Alliance approved US$ 85 million funding to support the introduction of typhoid conjugate vaccines in eligible countries in 2019–2020. 

bit.ly/2mmrDo4

## Insecticide resistance

Effective malaria prevention is threatened by widespread and increasing vector insecticide resistance. 

WHO has released new guidance for countries entitled *Framework for a national plan for monitoring and management of insecticide resistance in malaria vectors*. 

The new framework provides support for the development of a national insecticide resistance monitoring and management plan as part of a national malaria strategic plan.

The document outlines the content to be included and the key considerations to be taken into account when developing a national insecticide resistance monitoring and management plan. 

The guidance is designed to offer countries a framework that ensures adherence to the objectives and recommendations of the *Global plan for insecticide resistance management in malaria vectors*.

## Cholera in Zambia

The Zambian government launched a campaign last month to vaccinate residents of Lusaka with support from WHO and other partners, after an upsurge in cholera in December of last year. 

The campaign is part of a series of measures that have been taken since an outbreak was declared in October 2018.

Cholera is an acute diarrhoeal disease that can kill within hours if left untreated. Since the outbreak started in October 2017, the health ministry has reported 2672 cases and 63 deaths. Most of these cases (2558) and deaths (58) have been in the capital Lusaka.

Two million doses of the oral cholera vaccine (OCV) were released from the global stockpile that is funded by Gavi, the Vaccine Alliance, and delivered to the southern African country in early January, enough to immunize 1 million people.

“By giving 1 million people two doses each, rather than a single dose for 2 million people, we aim to gain the durable immunity that a two-dose regimen provides right away,” said Dr Nathan Bakyaita, the WHO Representative to Zambia. 

“The 2 million doses are part of a larger 4-million dose request to the global stockpile that would widen the use of OCV and make control measures more durable,” he said.

WHO is working with the Zambia National Public Health Institute to address the underlying causes of the cholera outbreak: clean water provision, sanitation and health education on personal hygiene.

Cholera cases are often reported in Zambia during the five-month rainy season, but the number of cases at the end of 2017 exceeded the average annual caseload. 

bit.ly/2EzyfHk

## Communicating risks in emergencies

Government authorities should provide clear public information on what is known and not known at a given time during outbreaks and other health emergencies, including the risks people face and how the situation is being addressed, according to a new WHO guideline.

Recent emergencies, such as the 2014–16 Ebola virus disease outbreak and the emergence of Zika virus syndrome in 2015–16, highlight the challenges of communicating health risks to the public.

The new guideline, released last month, was developed for people who manage emergencies, as well as frontline responders, development partners, and nongovernmental and private-sector organizations involved in emergency preparedness and response.

During public health emergencies, people need to know the health risks they face, and the actions to take to protect themselves, according to *Communicating risk in public health emergencies: a WHO guideline for emergency risk*.

The *International health regulations* (2005) requires governments to build capacity for communicating risk in health events. This new guideline provides advice on how to build such capacity. 

bit.ly/2r1hlj4

## WHO and UN Environment

UN Environment and WHO have agreed on a new collaboration to address the many environmental health risks that cause an estimated 12.6 million deaths a year.

The agreement signed last month covers joint action on air pollution, climate change and antimicrobial resistance as well as on waste and chemicals management, water quality, and food and nutrition issues. It also includes joint management of the BreatheLife campaign to reduce air pollution.

Under the new agreement, the two agencies will develop a joint work programme and hold an annual meeting to evaluate progress. 

bit.ly/2mvlwic

**Cover photo**

Cattle being transported in Welshpool, Powys, Wales in the United Kingdom of Great Britain and Northern Ireland.

**Figure:**
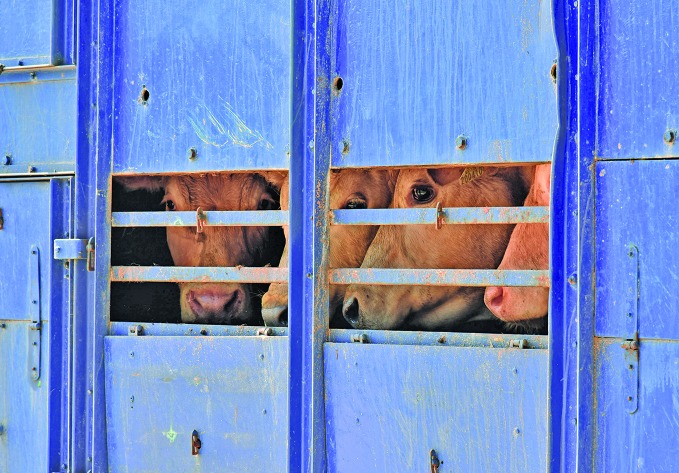
John Eveson

## Global dementia database

WHO has launched a web-based platform to track progress on the provision of services for people with dementia and for those who care for them, both within countries and globally.

The Global Dementia Observatory monitors whether countries have national policy and plans, as well as research that is being carried out and infrastructure provided for the provision of care and treatment.

The data and knowledge exchange platform, launched at the end of last year, also includes information on surveillance systems and the disease burden.

Currently nearly 10 million people develop dementia each year, 6 million of them in low- and middle-income countries. The number of people living with dementia is expected to triple from 50 million to 152 million by 2050. 

bit.ly/2qXwBxg

## Guidance on obstetric fistula

WHO issued a new recommendation last month on the length of bladder catheterization following surgical repair of a simple obstetric urinary fistula.

Currently the length of catheterization ranges from about 5 to 42 days. The new guidance recommends a 7 to 10-day period of bladder catheterization to allow for complete healing. The guidance is entitled *WHO recommendation on duration of bladder catheterization after surgical repair of simple obstetric urinary fistula*.

Long periods of catheterization are associated with discomfort for the woman as well as inconvenience for the woman, her family and care providers. 

bit.ly/2ATmeKo

## WHO and Global Fund 

WHO and the Global Fund to Fight AIDS, Tuberculosis and Malaria signed cooperation and financing agreements worth some US$ 50 million at the end of last year to fund collaboration in the fight against HIV, tuberculosis and malaria.

Specifically the agreements will underwrite WHO’s work with countries to increase access to pre-qualified medicines and other health products, develop and implement new funding applications, help find missing TB cases, improve data collection, accelerate action towards malaria elimination in 21 countries and pilot the introduction of the RTS,S malaria vaccine in Ghana, Kenya and Malawi.

WHO and the Global Fund are also developing a framework of strategic collaboration to guide their institutional relationship. 

bit.ly/2D59cj8

## Technologies for TB medication 

WHO released a new handbook last month on three technologies that can be used to help tuberculosis patients all over the world complete their treatment over the many months that their drug regimens last.

The three technologies are: short message service (SMS), medication event monitoring systems (MEMS) and video-supported treatment (VOT).

The handbook, entitled *WHO handbook for the use of digital technologies to support tuberculosis medication adherence*, was produced by WHO in collaboration with the European Respiratory Society (ERS), other technical partners and national tuberculosis programme staff. 

It is aligned with the WHO/ERS digital health agenda for action for tuberculosis and evidence-based recommendations on the use of SMS, MEMS, VOT and other digital technologies released by WHO in 2017. bit.ly/2AS65VL

**Looking ahead**

7 April – World Health Day devoted to the subject of universal health coverage

24–30 April – World Immunization Week

21–26 May – Seventy-first World Health Assembly

31 May – World No Tobacco Day

